# Accurate image reconstruction within and beyond the field-of-view of CT system from data with truncation

**DOI:** 10.1088/1361-6560/ada7be

**Published:** 2025-01-27

**Authors:** Zheng Zhang, Buxin Chen, Dan Xia, Emil Y Sidky, Xiaochuan Pan

**Affiliations:** 1 Department of Radiology, The University of Chicago, Chicago, IL 60637, United States of America; 2 Department of Radiation and Cellular Oncology, The University of Chicago, Chicago, IL 60637, United States of America

**Keywords:** computed tomography, truncation, total variation, ℓ_1_-norm, optimization, primal-dual algorithm

## Abstract

*Objective*. Accurate image reconstruction from data with truncation in x-ray computed tomography (CT) remains a topic of research interest; and the works reported previously in the literature focus largely on reconstructing an image only within the scanning field-of-view (FOV). We develop algorithms to invert the truncated data model for numerically accurate image reconstruction within the subject support or a region slightly smaller than the subject support. *Methods*. We formulate image reconstruction from data with truncation as an optimization program, which includes hybrid constraints on region-based image total-variation (TV) and image $\ell_1$-norm (L1) for effectively suppressing truncation artifacts. An algorithm, referred to as the TV-L1 algorithm, is developed for image reconstruction (i.e. inversion of the data model) from data with truncation through solving the optimization program. *Results*. We perform numerical studies to evaluate accuracy and stability of the TV-L1 algorithm by using simulated and real CT data. Accurate images can be obtained stably by use of the TV-L1 algorithm within the subject support, or a region substantially larger than the FOV, from data with truncation of varying degrees. *Conclusions*. The TV-L1 algorithm can invert the truncated data model to accurately and stably reconstruct images within the subject support, or a region slightly smaller than the subject support but substantially larger than the FOV. *Significance*. Accurate image reconstruction within the subject support, or a region substantially larger than the FOV, from data with truncation can be of theoretical and practical implication. The insights and TV-L1 algorithm may also be generalized to accurate image reconstruction from data with truncation in other tomographic imaging modalities.

## 1. Introduction

In x-ray computed tomography (CT), the scanning field-of-view (FOV) includes a collection of points in the image space that are visible from each of the scanning views. Data truncation occurs when the FOV cannot completely cover the subject support at each, or some, of the scanning views. It may be encountered in certain CT imaging applications, such as image-guided interventional procedures (Daly *et al* 1999, Paulson *et al* 2001, Hirota *et al* 2006, Lauritsch *et al* 2006, Wallace *et al* 2008, Solomon and Silverman 2010), image-guided surgery (Hott *et al* 2004, Siewerdsen *et al* 2005, Schafer *et al* 2011), image-guided radiation therapy (Létourneau *et al* 2005, Oldham *et al* 2005, Tognolini *et al* 2010, Floridi *et al* 2014, Bapst *et al* 2016), and non-destructive testing (Zhang *et al* 2015, Kim *et al* 2021), where data collected can be truncated as the scanning FOV is smaller than the subject support, and/or as a narrow beam is used often for illuminating only a region-of-interest (ROI) within the subject support for lowering radiation dose. Image artifacts can be observed within and outside the FOV reconstructed from data with truncation by use of some of the existing algorithms; and they can bias image values and prevent image details of interest from being appropriately visualized (Hsieh 2003, Boas *et al* 2012).

Conventional algorithms such as the filtered backprojection (FBP) algorithm (Natterer and Wübbeling 2001, Hsieh 2003), when applied to data with truncation, yield images with visual artifacts and biased image values. Local-tomography algorithms (Kuchment *et al* 1995, Faridani *et al* 1997, Anastasio *et al* 2007, Quinto *et al* 2009) may yield image discontinuities (i.e. image edges) within the FOV from truncated data. However, there remains a strong interest in, and significant needs for, accurately reconstructing image values from data with truncation. Algorithms have been developed for reconstructing images on truncated chords that form the image within the FOV (Zou *et al* 2004, Defrise *et al* 2006, Kudo *et al* 2008, Tang *et al* 2012). Other algorithms, including optimization-based algorithms (Yang *et al* 2010, Taguchi *et al* 2011, Lauzier *et al* 2012, Xia *et al* 2016), have also been investigated for image reconstruction from data with truncation.

Studies have been reported in which an image is reconstructed within an ROI larger than the FOV through empirical extrapolation of data collected to cover the ROI (Ohnesorge *et al* 2000, Hsieh *et al* 2004, Sourbelle *et al* 2005, Huang *et al* 2021, Podgorsak *et al* 2021, Khural *et al* 2022). However, the accuracy of data extrapolated, and thus of the image reconstructed, by use of the empirical extrapolation approach diminishes rapidly, as the truncation degree and/or the complexity level of the subject anatomy missed in truncated data, increase.

To the best of our knowledge, no algorithms reported previously can effectively invert the truncated data model for numerically accurate and stable image reconstruction within the subject support, or within a region slightly smaller than the subject support but still substantially larger than the FOV from data with truncation. Thus, the investigation and development of such algorithms, which are the focus of the work, remain of theoretical interest and of practical implication.

The work is inspired by the observations made in the analysis of the root cause of truncation artifacts illustrated in appendix A. While the analysis is done for a parallel-beam geometry for simplicity and clarity, the analysis and insight gained can be directly applicable to the analysis of truncation artifacts in fan- and cone-beam geometries. In a parallel-beam scan over 180^∘^, a necessary condition on the accurate reconstruction of a pixel value is that the directions of measurable x-rays passing through the pixel span the angular range of 180^∘^. Conversely, the directions of measurable x-rays in the presence of data truncation passing through a pixel outside the FOV span only limited-angular ranges (LARs) whose summation is smaller than 180^∘^; and the starting angles and extents of the LARs also vary for pixels at different locations outside the FOV. Therefore, a truncation problem is in essence tantamount to a pixel-varying-LAR problem yielding artifacts of characteristics analogous to that observed in images reconstructed from LAR data (Zhang *et al* 2021).

We formulate the reconstruction problem from truncated data (or, equivalently, the inverse problem of the truncated data model) as an optimization program, which includes hybrid, region-based image total-variation (TV) and $\ell_1$-norm (L1) constraints for effective suppression of truncation artifacts in images reconstructed. An algorithm, referred to as the TV-L1 algorithm, is developed subsequently for inverting the truncated data model to yield numerically accurate and stable image reconstruction within the subject support or a region slightly smaller than the subject support, which is substantially larger than the FOV by solving the optimization program.

Numerical studies are conducted for assessing the accuracy and stability of the TV-L1 algorithm for image reconstruction from simulated and real data with truncation of varying degrees. The study results demonstrate that the TV-L1 algorithm can invert the truncated data model for yielding numerically accurate and stable image reconstruction within the subject support or a region slightly smaller than the subject support, yet substantially larger than the FOV, from simulated and real data with truncation of varying degrees.

The TV-L1 algorithm is developed first in section 2, whereas numerical studies are carried out using simulated data for empirically investigating the algorithm potential in section 3, and using real data to demonstrate the algorithm stability in section 4, for image reconstruction from truncated data of varying degrees. We discuss and conclude the work in sections 5 and 6.

## 2. Methods

### 2.1. Truncated scanning configurations

**Figure 1.**
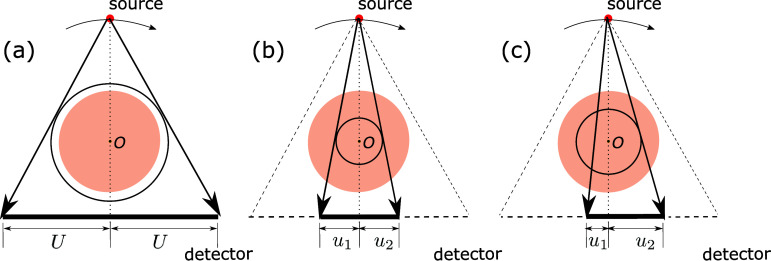
Schematics of fan-beam scanning configurations with linear detectors of varying lengths in each of which the central line (dotted) connecting the center-of-rotation (COR) *O* and the x-ray source (red dot) is perpendicular to the linear detector. Using $u_1 > 0$ and $u_2 > 0$ to denote the detector lengths on both sides of the central line, we consider configurations (a) of $u_1 = u_2 = U$, (b) of $u_1 = u_2 < U$, and (c) of $u_1 < u_2$ & $u_1+u_2 < U$, forming FOVs specified, respectively, by the circles (black), where 2*U* is the full detector length. We use the disk (cantaloupe) to depict the support of the subject scanned and assume that the centers of the subject support and FOV coincide with COR *O*. In (a), the subject support is enclosed completely within the FOV, resulting in no data truncation, whereas in (b) and (c), FOVs are enclosed completely, instead, within the subject support, thus resulting in data truncation. We refer to (a), (b), and (c), respectively, as the non-truncated, symmetrically truncated, and asymmetrically truncated configurations. When configurations (b) and (c) are of identical length $u_1+u_2$, the FOV of (c) is thus larger than that of (b). Dimensionless *U*, *u*_1_, and *u*_2_ denote the numbers of detector bins, and the physical size of a detector bin is specified in each of the numerical studies in sections 3 and 4 below.

In the work, the fan-beam scanning configurations displayed in figure 1 are considered, with the understanding that the concepts, analysis, and algorithm developed in the work can be extended straightforwardly to cone-beam scanning configurations. It can be observed that each of the configurations in figure 1 includes a linear detector and an x-ray point source, center-of-rotation (COR) *O*, and a central line connecting *O* and the source point is perpendicular to the linear detector. In this work, we assume that the subject scanned is of a finite support enclosed completely within the FOV of a configuration of full length 2*U*, as displayed in figure 1(a). Also, using $u_1\in (0, U)$ and $u_2\in (0, U)$ to denote the detector lengths on both sides of the central line, we obtain a symmetrically truncated configuration of $u_1 = u_2$ in figure 1(b) and an asymmetrically truncated configuration of $u_1 < u_2$ in figure 1(c), where $u_1 + u_2 < U$. Without loss of generality, the centers of the subject support and the FOV coincide with COR *O* of the scanning configuration.

We generate simulated data or collect real data over 2*π* of a circular trajectory with configurations in figure 1, and note that its extension to a non-circular trajectory is trivial. In each of the configurations in figure 1, the disk (cantaloupe) denotes the subject support, whereas the circle (black) depicts the FOV of the configuration. The FOV of a detector of full length in figure 1(a) completely encompasses the subject support, generating or collecting data without truncation, whereas the FOVs in figures 1(b) and (c) are enclosed completely by the subject support, incurring data truncation. For convenience, we interchangeably use below ‘subject support’ and ‘image support’.

### 2.2. Data model with truncation

For a scanning configuration displayed in figure 1, data are collected on a discrete data array, whereas the image is discretized on an array of square-shaped pixels. We use vector **g** of size $J_{u_1 u_2}$ to represent model data in a concatenated form in the order of rays within each projection view and then projection views, with entry *g_j_* denoting measurement of ray *j*, $j = 1, 2, {\ldots}, J_{u_1 u_2}$, where $J_{u_1 u_2} = J^{[d]}_{u_1 u_2}\times J^{[\phi]}$ is the total number of rays measured that pass through FOV, $J^{[d]}_{u_1 u_2}$ the number of detector bins at each view, and $J^{[\phi]}$ the total number of views. Also, let vector **f** of size *I* denote the image in a concatenated form in the order of *x* and *y*, with entry *f_i_* indicating image value at pixel *i*, $i = 1, 2, {\ldots}, I$, and *I* the size of the image array. The truncated data model is written as

\begin{eqnarray*} \mathbf{g} = \mathcal{H}^{\left(u_1, u_2\right)} \mathbf{f},\end{eqnarray*}

where matrix $\mathcal{H}^{(u_1, u_2)}$ of size $J_{u_1 u_2}\times I$ denotes a 2D discrete x-ray transform with truncation specified by *u*_1_ and *u*_2_, as depicted in figure 1. Element *h*_*ji*_ of matrix $\mathcal{H}^{(u_1, u_2)}$ is chosen to be the weighted intersection length of ray *j* with pixel *i*. When $u_1 = u_2 = U$ in figure 1(a), $\mathcal{H}^{(U, U)}$ becomes the standard, 2D discrete x-ray transform without truncation of size $J_{U U}\times I$. Image reconstruction from data with truncation is tantamount to inverting the truncated data model in equation (1). Depending upon the truncation degree, $\mathcal{H}^{(u_1, u_2)}$ can be severely ill-posed.

### 2.3. Image regions for specifying constraints

**Figure 2.**
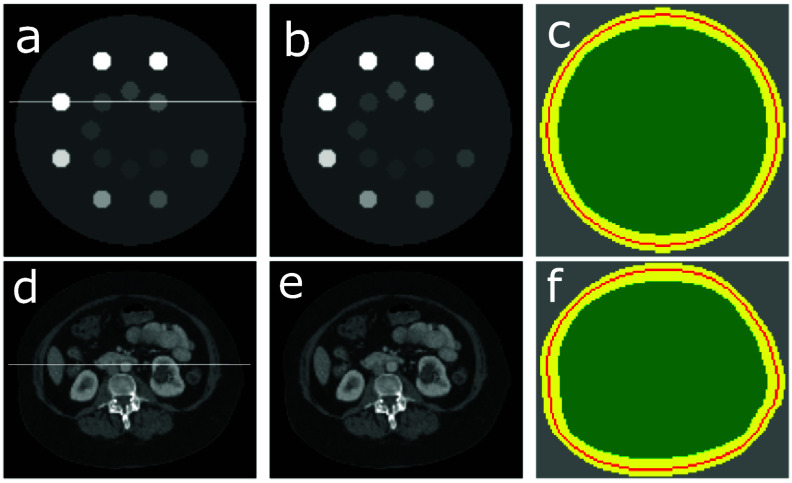
(a) and (d): Digital DE and abdomen phantoms; (b) and (e): the images of the digital DE and abdomen phantoms reconstructed within their respective supports by use of the TV-L1 algorithm from simulated data without truncation, display windows: [0.1, 0.3] cm^−1^; and (c) and (f): schematics of annulus regions $\mathcal{M}_o $ (yellow) containing the exterior boundaries (red contours) and inner regions $\mathcal{M}_i $ (green) of the digital DE and abdomen phantoms. Image profiles are plotted over the white horizontal lines drawn in (a) and (d) in the simulated-data study below.

As shown below, the reconstruction problem (or, equivalently, the inverse problem of the truncated data model) is formulated as an optimization program with hybrid $\ell_1$-norm and TV constraints on selected image regions. Let masks $\mathcal{M}_o$ and $\mathcal{M}_i$ be diagonal matrices of size *I* × *I* with diagonal elements that can be 0 or 1. When applied to the image array, $\mathcal{M}_o$ selects a annulus region containing the exterior boundary of the image support, whereas $\mathcal{M}_i$ selects the region within the image support inscribed in the interior boundary of the annulus region of $\mathcal{M}_o$, and thus $\mathcal{M}_o$ selected completely specifies region $\mathcal{M}_i$. In figures 2(c) and (f), we display schematically the exterior boundaries (red) contained within the annulus regions (yellow) of $\mathcal{M}_o$, along with regions (green) of $\mathcal{M}_i$, selected for the digital DE and abdomen phantoms. As discussed below, we devise unique TV and $\ell_1$-norm constraints on images within region $\mathcal{M}_i$ (green) and annulus region $\mathcal{M}_o$ (yellow), respectively, for tightening the feasible solution set of the optimization program formulated below.

Mask $\mathcal{M}$ of size *I* × *I* is introduced further for selecting a region that is the union of the regions of $\mathcal{M}_o$ and $\mathcal{M}_i$ completely, tightly enclosing the image support; and we use it in the optimization program for assigning image values of 0 to pixels outside region $\mathcal{M}$.

### 2.4. Optimization program with hybrid image constraints

Considering mask $\mathcal{M}$ discussed above, we have $\mathbf{f} = \mathcal{M} \mathbf{f}$ and can thus re-express the truncated data model in equation (1) as

\begin{eqnarray*} \mathbf{g} = \mathcal{H}^{\left(u_1, u_2\right)}\mathcal{M} \mathbf{f}.\end{eqnarray*}

Image reconstruction now is tantamount to the inversion of the truncated data model in equation (2), and we formulate the reconstruction problem from data with truncation (or, equivalently, the inverse problem of the truncated data model) as a convex optimization program. Clearly, depending upon the degree of truncation, the truncated data model can be highly ill-posed, resulting in a sizable feasible solution set suited to data with truncation given. Therefore, constraints are introduced in the optimization program for tightening the feasible solution set.

Instead, we propose the image $\ell_1$-norm and TV defined, respectively, on regions $\mathcal{M}_0$ and $\mathcal{M}_i$, which together form a complementary partition of the subject support. It is the hybrid constraint on the region-based image $\ell_1$-norm and TV that may allow for a refined exploitation of the image sparsity and its gradient sparsity in different regions for yielding numerically accurate image reconstruction, as the numerical results evidenced in the manuscript: (a) because $\mathcal{M}_0$, the narrow annulus region, contains the exterior boundary of the subject support, the image itself within annulus region $\mathcal{M}_0$ should be sparse, and the image $\ell_1$-norm defined on annulus region $\mathcal{M}_0$ is used for promoting pixel sparsity within annulus region $\mathcal{M}_0$ and hence for containing leakage outside of the exterior boundary of the subject; and (b) conversely, because region $\mathcal{M}_i$ is interior to annulus region $\mathcal{M}_0$, the image TV defined only on region $\mathcal{M}_i$ can avoid the significant contribution from image within annulus region $\mathcal{M}_0$, thus allowing for promoting a tighter TV (i.e. gradient-sparsity) constraint and hence a tighter feasible solution set.

Including the region-based TV and $\ell_1$-norm constraints, we formulate the optimization program as

\begin{align*} \mathbf{f}^{\star} &amp; = \underset{\mathbf{f}}{\mathsf{argmin}} \left\{\frac{1}{2} \parallel \mathcal{W} \left(\mathcal{H} ^{\left(u_1, u_2\right)}\mathcal{M} \mathbf{f} - \mathbf{g}^{\left[\mathcal{M}\right]}\right) \parallel_2^2 \right\} \nonumber \\ &amp; \qquad\textrm{s.t.} \,\, || \mathcal{M}_o \mathcal{M} \mathbf{f} ||_1 \unicode{x2A7D} l, \,\, ||\left(|\mathcal{M}^{\prime}_i \nabla \mathcal{M} \mathbf{f}|\right) ||_1 \unicode{x2A7D} t, \,\,\textrm{and} \,\, f_i \unicode{x2A7E} 0,\end{align*}

where vector $\mathbf{g}^{[\mathcal{M}]}$ of size $J_{u_1 u_2}$ denotes measured data; $||\cdot||_2^2$ the data fidelity term in the $\ell_2$-norm form; $|| \mathbf{x} ||_1$ the image $\ell_1$-norm, along with $\ell_1$-constraint parameter *l*; $\nabla$ the gradient matrix of size $2I \times I$ of form $\nabla^\top = (\nabla_x^\top, \nabla_y^\top)$; → *p* the transpose operation; $\nabla_x$ and $\nabla_y$ matrices of size *I* × *I*, representing two-point differences along *x* and *y* axes, respectively; $|| (|\nabla \mathbf{x}|) ||_1$ the image TV (Sidky and Pan 2008), $\mathcal{M}^{^{\prime}}_i$ a diagonal matrix of size $2I\times 2I$ satisfying

\begin{eqnarray*} \mathcal{M}^{\prime}_i = \begin{pmatrix} \mathcal{M}_i \quad \mathbf{0} \nonumber \\ \mathbf{0} \quad \mathcal{M}_i \nonumber \\ \end{pmatrix} , \quad\quad \mathcal{M}^{\prime}_i \nabla \mathcal{M} \mathbf{f} = \begin{pmatrix} \mathcal{M}_i \nabla_x \mathcal{M} \mathbf{f} \nonumber \\ \mathcal{M}_i \nabla_y \mathcal{M} \mathbf{f} \nonumber \\ \end{pmatrix};\end{eqnarray*}

**0** a matrix of size *I* × *I* with all elements of 0; $\mathcal{W}$ is a diagonal matrix of size $J_{u_1 u_2}\times J_{u_1 u_2}$, containing $J^{[\phi]}$ blocks of identical diagonal matrix $\mathcal{W}^{[d]}$ of size $J^{[d]}_{u_1 u_2}\times J^{[d]}_{u_1 u_2}$, with diagonal elements $w^{[d]}_{j_{_d} j_{_d}}$, where $j_{_d} = 1, 2, {\ldots}, J^{[d]}_{u_1 u_2}$. For a symmetrical truncation configuration, $w^{[d]}_{j_{_d} j_{_d}} = 1$; whereas for an asymmetrical truncation configuration, $w^{[d]}_{j_{_d} j_{_d}}$ is chosen to be the Parker weighting factor (Parker 1982) given by

\begin{eqnarray*} w^{\left[d\right]}_{j_{_d} j_{_d}} = \begin{cases} \textrm{cos}^2 \frac{\pi}{4} \left(\frac{\tau_{j_{_d}}}{\tau_c} - 1\right) \quad \textrm{for} \quad 0 \unicode{x2A7D} j_{_d} \unicode{x2A7D} 2 u_1\\ 1 \quad\quad\qquad\qquad\quad \textrm{for} \quad j_{_d} > 2 u_1 \end{cases};\end{eqnarray*}

where $\tau_c = \textrm{atan}\frac{u_1\Delta_u}{S}$; $\tau_{j_{_d}} = \textrm{atan}\frac{({j_{_d}}-u_1)\Delta_u}{S}$; $\Delta_u$ denotes the size of a detector bin; and *S* the source to detector distance.

We use $\mathcal{W}$ to normalize possible redundant information within data collected over 2*π*. For a symmetrically truncated configuration of $u_1 = u_2$, information in data collected by using the *u*_1_ and *u*_2_ detector bins is completely redundant, and the use of $w^{[d]}_{j_{_d} j_{_d}} = 1$ automatically normalizes the complete data redundance. Conversely, for an asymmetrically truncated configuration of $u_1 < u_2$, information collected by use of the *u*_1_ detector bins is redundant only partially with that collected by using the *u*_2_ detector bins, and equation (4) thus normalizes the partial data redundance. It is well known that the Parker weighting factor specified in equation (4) can be exploited for suppressing image artifacts reconstructed from data containing various physical factors in a real-data study (Bian *et al* 2012).

It is well known that any approaches to image reconstruction involve parameters. Three tunable constraint parameters $\mathcal{M}_o$, *l*, and *t* are used in equation (3) for specifying the feasible solution set of the optimization program; and different selections (i.e. the tuning) of the constraint parameters lead to different feasible solution sets and thus different image reconstructions from the same data (Zhang *et al* 2016). Therefore, accurate image reconstruction depends upon appropriate selections (i.e. tuning) of the constraint parameters, which are discussed specifically in each of the simulated- and real-data studies in sections 3 and 4 below.

### 2.5. TV-L1 algorithm for image reconstruction

We develop the TV-L1 algorithm, a primal-dual-based algorithm (Rockafellar 1999, Chambolle and Pock 2011, Sidky *et al* 2012) to solve the convex optimization program in equation (3) and display its pseudo-codes in algorithm 1. In the pseudo-codes, input information includes measured data $\mathbf{g}^{[\mathcal{M}]}$, system matrix $\mathcal{H}^{(u_1, u_2)}$, and tunable parameters $\mathcal{M}_o$, *l*, and *t*, along with algorithm parameter *ρ* > 0 that can be chosen for empirically optimizing the convergence rate of the TV-L1 algorithm to the feasible solution set. It is noted that $\mathcal{M}_i$ is specified completely by $\mathcal{M}_o$ selected in the TV-L1 algorithm. Moreover, $\mathcal{T}$ and Σ are non-diagonal and diagonal matrices of size *I* × *I*, which can be computed as

\begin{eqnarray*} \mathcal{T} &amp; = &amp; \frac{1}{\rho} \left( \frac{\mathcal{I}}{e_K} + \sum_{k = 1}^{K-1} \mathbf{x}_k \left(\frac{1}{e_k} - \frac{1}{e_K}\right) \mathbf{x}_k^{\top} \right) \quad \textrm{and}\quad \Sigma = \rho \varsigma \mathcal{I},\end{eqnarray*}

where $\mathcal{I}$ denotes an identity matrix of size *I* × *I*; *e_k_* and **x**_*k*_ the *k*th largest eigenvalue and corresponding eigenvector of matrix $(\mathcal{W}{\mathcal{H}}^{(u_1, u_2)})^{\top} (\mathcal{W}\mathcal{H}^{(u_1, u_2)})$, *K* the number of eigenvectors in the expansion and *K* = 10 is used in the work; $\varsigma = {||\mathcal{T} ({\mathcal{K}}^\top {\mathcal{K}})||_2}^{-1}$; $\mathcal{K}^{\top} = (\mathcal{W}{\mathcal{H}}^{(u_1, u_2)\top}, \nu_1 \mathcal{M}^\top \nabla^\top \mathcal{M}^{^{\prime}\top}_i$, $\nu_2 \mathcal{M}^\top \mathcal{M}^\top_o$, $\mu\mathcal{I}$). Furthermore, $||\cdot||_2$ denotes the largest singular value of a matrix; vector $\mathbf{w}^{(n)}$ is of size *J*; vectors $\bar{\mathbf{p}}^{(n)}$ and $\mathbf{p}^{(n)}$ are of size 2*I*; vectors $\bar{\mathbf{q}}^{(n)}$, $\mathbf{q}^{(n)}$, and $\mathbf{t}^{(n)}$ are of size *I*; operator $\textrm{neg}(\cdot)$ enforces the non-positivity constraint; operator $\ell_1 \textrm{ball}_{\omega}(\cdot)$ projects a vector onto the $\ell_1$-ball of scale *ω*; $|\bar{\mathbf{p}}^{(n)}|$ and $|\bar{\mathbf{q}}^{(n)}|$ depict vectors of size *I* with entry *i* given by $(|\bar{\mathbf{p}}^{(n)}|)_i = \sqrt{(\bar{p}^{(n)}_i)^2 + (\bar{p}^{(n)}_{i+I})^2}$ and $(|\bar{\mathbf{q}}^{(n)}|)_i = |\bar{q}^{(n)}_i|$, respectively; and $\bar{p}^{(n)}_i$ and $\bar{q}^{(n)}_i$ indicate the *i*th entry of vectors $\bar{\mathbf{p}}^{(n)}$ and $\bar{\mathbf{q}}^{(n)}$, respectively.

### 2.6. Evaluation schemes and metrics

We first perform a qualitative evaluation of possible truncation artifacts through visual inspection of images reconstructed, followed by semi-quantitative analysis of image profiles reconstructed relative to the truth or reference image profiles. Finally, we use goodness metrics, the normalized root-mean-square error (nRMSE) and Pearson-correlation coefficient (PCC) (Bian *et al* 2010), summarized in appendix B to quantitatively evaluate reconstruction accuracy of the TV-L1 algorithm. In the simulated- and real-data studies in sections 3 and 4, we also include reconstruction results of the standard FBP algorithm with the Shepp-Logan filter simply for demonstrating the possible reduction of truncation artifacts by use of the TV-L1 algorithm under the truncation conditions considered.

## 3. Simulated-data studies

We perform studies using simulated data for two folds of purpose: (1) to numerically verify first that the TV-L1 algorithm can numerically accurately invert the data model with no truncation from simulated data like what many existing algorithms can do; and (2) to numerically survey then the potential (i.e. the performance upper bound) of the verified TV-L1 algorithm for inverting data model in equation (2) with truncation of varying degrees to yield numerically accurate image reconstruction within the support of the phantom that is substantially larger than the FOV from data with truncation of varying degrees.

The stability of the TV-L1 algorithm in the presence of physical factors, such as noise, scatter, and beam-hardening (Pan *et al* 2009) that are inconsistent with (i.e. are not included in) data model equation (2), is studied numerically in section 4 by using real data that contain these physical factors. While we have also studied numerically the TV-L1 algorithm by using simulated data containing noise, we opt not to include the study results because they do not reveal knowledge more than that acquired in the real-data studies in section 4.

### 3.1. Experimental design of the simulated-data studies

#### 3.1.1. Digital phantoms

In the simulated-data studies, we consider the digital DE and abdomen phantoms of distinctly different anatomies, as shown in figures 2(a) and (d). The DE phantom mimics the physical DE phantom that is used widely for CT imaging calibration (JRTAssociates 2015), whereas the digital abdomen phantom is obtained from a clinical CT image of the human abdomen (Yu *et al* 2012, Chen *et al* 2015). Each of the digital phantoms is embedded in a 2D array of $128\times 128$ square pixels, each of which is of size 2.7 mm.

#### 3.1.2. Scanning configuration geometry

The fan-beam scanning configurations with a circular trajectory depicted in figure 1 are considered in the simulated-data studies, which have source-to-rotation distance (SRD) and source-to-detector distance (SDD) of 100 cm and 150 cm, whereas the full length of the linear detector composes $2 U = 380$ detector bins of size 1.4 mm, yielding a fan angle of 20.1^∘^.

#### 3.1.3. Data generation

Using each of the digital phantoms in data model equation (1) with $u_1 = u_2 = U$ bins, we first generate full data without truncation at 360 views uniformly distributed over 2*π* by using the scanning configuration in figure 1(a). Subsequently, we extract, from full data, data with truncation of varying degrees at each of 360 views for each of the symmetrically truncated configurations listed in table 1.

**Table 1.** Symmetrically truncated configurations in the simulated-data studies of the digital DE and abdomen phantoms, with $2U = 380$ detector bins.

Configurations	$(u_1, u_2)$	$(u_1 + u_2)$	$(u_1+u_2)/2U$
A	(150, 150)	300	78.9%
B	(100, 100)	200	52.6%
C	(80, 80)	160	42.1%
D	(60, 60)	120	31.6%
E	(40, 40)	80	21.1%
F	(20, 20)	40	10.5%

#### 3.1.4. Selection (i.e. tuning) of the constraint parameters

Any algorithms for image reconstruction must involve parameters. As discussed above, the optimization program in equation (3) involves tunable constraint parameters $\mathcal{M}_o$, *l*, and *t* that need to be selected (i.e. tuned) prior to a reconstruction. In our simulated-data study, we have full knowledge of the digital phantom, such as its support and thus exterior boundary, from which truth $\mathcal{M}_o$, *l*, and *t* can readily be obtained. As our goal is first to verify the TV-L1 algorithm with non-truncated data and then to reveal its performance upper bounds in reconstruction from truncated data, we thus use the truth constraint parameters obtained from the digital phantom.

Specifically, we select $\mathcal{M}_o$ as an annulus region (yellow) of three-pixel width that contains the exterior boundary (red) of the digital DE or abdomen phantom, as shown in figures 2(c) or (f). With $\mathcal{M}_o$ selected, $\mathcal{M}_i$ is determined completely that specifies a region inscribed in the interior boundary of the annulus region of $\mathcal{M}_o$. Furthermore, $\ell_1$-norm and TV constraint parameters *l* and *t* can readily be computed within the annulus region (yellow) of $\mathcal{M}_o$ and region (green) of $\mathcal{M}_i$. (We note that in figures 2(c) or (f), the annulus region (yellow) of a width larger than the three-pixel width is displayed schematically for clearly revealing the exterior boundary (red) of the digital phantom.)

In the absence of full knowledge of a subject imaged in, e.g. real-data studies, truth knowledge of the subject is unavailable. Therefore, as described in section 4 below, in a real-data study, we first perform multiple reconstructions with different sets of the constraint parameters chosen and then select the set of the constraint parameters that yields the empirical ‘maximum’ of a qualitative or quantitative goodness metric used for guiding the selection of the constraint parameters.

#### 3.1.5. Image analysis

In each of the simulated-data studies below, the final image is reconstructed when the necessary convergence conditions in appendix B are reached numerically in terms of single-precision or double-precision floating-point error (Chambolle and Pock 2011, Sidky *et al* 2012, Zhang *et al* 2021). We first perform visual inspection of the image reconstructed within the support of the digital phantom, followed by a numerical analysis of the image profiles reconstructed. Using the digital DE or abdomen phantom as the reference image in equations (B.3) and (B.5), we also compute quantitative metrics nRMSE and PCC from the images reconstructed within the support of the digital phantom.

### 3.2. Results of the digital DE phantom

In each simulated-data study of the digital DE phantom, we select $\mathcal{M}_o$ and compute *l* and *t*, as described in section 3.1 above. With the parameters selected, we first perform a study to verify that the TV-L1 algorithm (and its computer implementation) can indeed reconstruct, from full data without truncation generated, an image that is numerically identical to the digital DE phantom (i.e. in terms of the single-precision floating-point error). The quantitative study reveals that the image reconstructed in figure 2(b) is indeed numerically identical to the digital DE phantom in figure 2(a). We then use the TV-L1 algorithm verified to investigate below its potential for numerically accurate image reconstruction of the digital DE phantom from data generated with the symmetrically truncated configurations listed in table 1.

#### 3.2.1. Visual inspection of the images reconstructed

**Figure 3.**
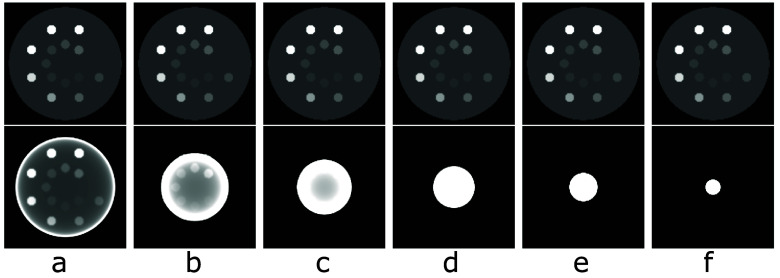
Images of the digital DE phantom reconstructed within the phantom support by use of the TV-L1 (row 1) and FBP (row 2) algorithms from simulated data of the phantom generated with symmetrically truncated configurations A (a), B (b), C (c), D (d), E (e), and F (f) listed in table 1. Display window: [0.1, 0.3] cm^−1^.

Images of the digital DE phantom reconstructed within the phantom support by use of the TV-L1 and FBP algorithms are displayed, respectively, in rows 1 and 2 of figure 3. It can be seen that the TV-L1 algorithm yields visually accurate images within the support of the digital DE phantom for all of the symmetrically truncated configurations listed in table 1 and that significant truncation artifacts observed in the FBP reconstructions are corrected for in the images of the TV-L1 algorithm.

#### 3.2.2. Quantitative evaluation of the images reconstructed

**Figure 4.**
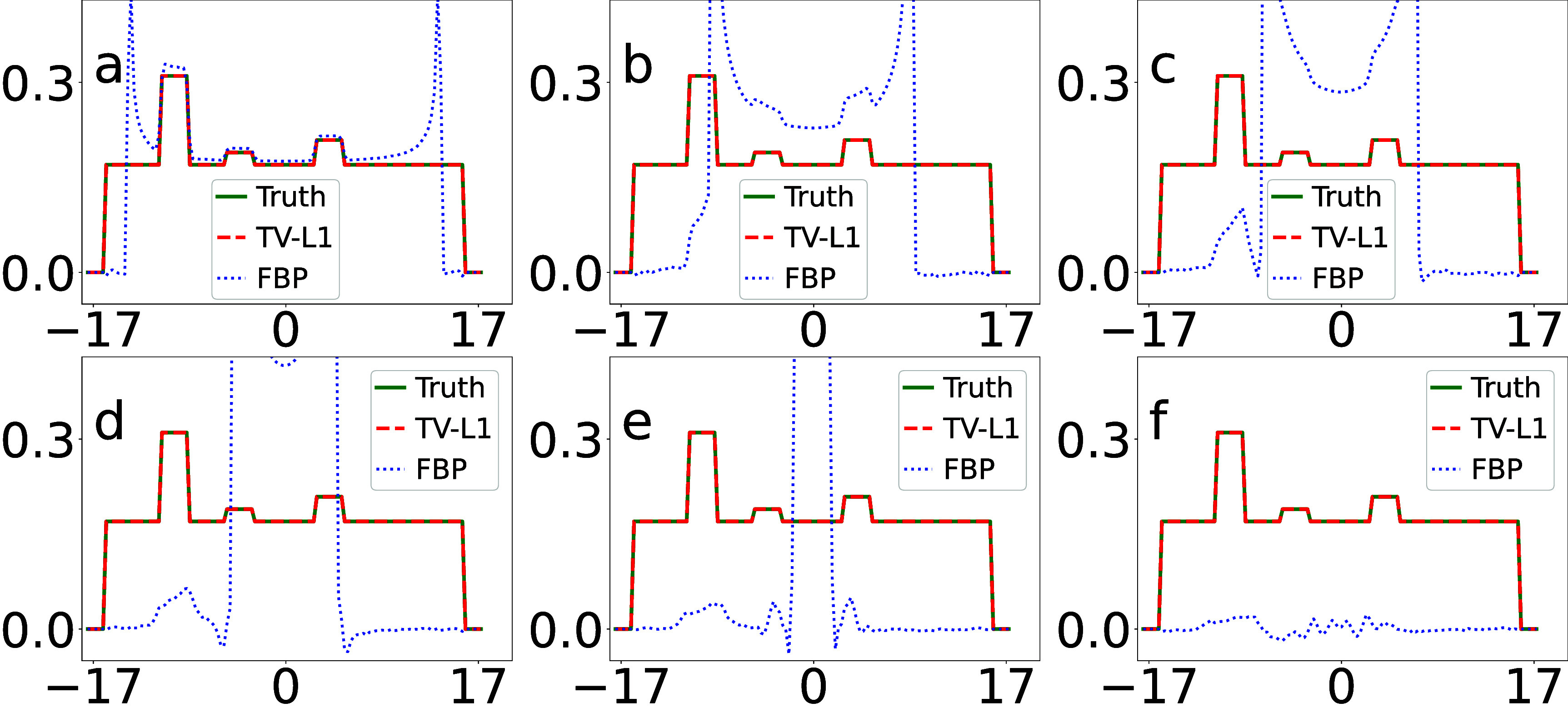
Image profiles (a)–(f) of the digital DE phantom in figure 3 over the white line indicated in figure 2(a), reconstructed by use of the TV-L1 (dashed) and FBP (dotted) algorithms, along with the corresponding truth image profiles (solid) of the digital DE phantom. The abscissas in the plots are of unit cm. We note that the image-profile values of the FBP reconstructions can be beyond the maximum value used in the plot.

We display in figure 4 the profiles of the images in figure 3 over the white horizontal line highlighted in figure 2(a) for revealing quantitatively image-reconstruction accuracy. It can be observed that the image profiles of the TV-L1 reconstruction from data with truncation of varying degrees agree well with their respective truth profiles, correcting for the significant biases of the image profiles in the FBP reconstructions in figure 4.

**Figure B2.**
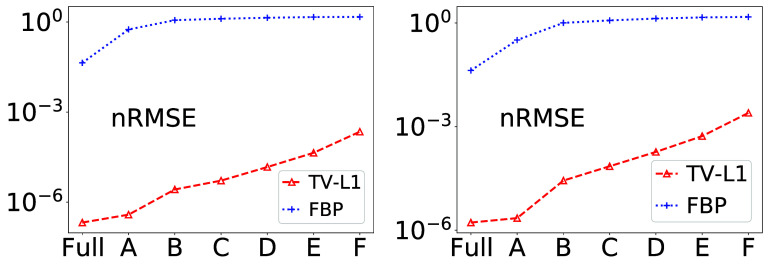
Metric nRMSE calculated from the images of the digital DE phantom (left) and digital abdomen phantom (right) reconstructed by use of the TV-L1 (dashed) and FBP (dotted) algorithms.

We compute metrics nRMSE and PCC from images of the digital DE phantom in figure 3 and display them in figures B2 and B4, which reveal that as the level of data truncation increases, while metric nRMSE increases moderately, metric PCC remains largely 1, indicating numerically accurate image reconstruction of the digital DE phantom.

### 3.3. Results of the digital abdomen phantom

In each simulated-data study of the digital abdomen phantom, $\mathcal{M}_o$ is selected, and *l* and *t* are computed, as described in section 3.1 above. With the parameters selected, we again perform a study verifying that the TV-L1 algorithm can reconstruct, from full data without truncation, an image that is numerically identical to the digital abdomen phantom, as shown in figure 2(e). We then use the TV-L1 algorithm verified to investigate below its potential for numerically accurate image reconstruction of the digital abdomen phantom from data generated with the symmetrically truncated configurations listed in table 1.

#### 3.3.1. Visual inspection of the images reconstructed

**Figure 5.**
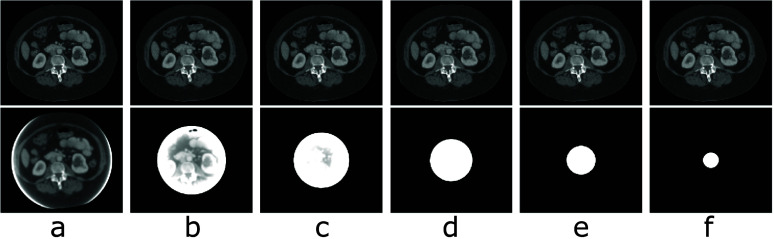
Images of the digital abdomen phantom reconstructed within the phantom support by use of the TV-L1 (row 1) and FBP (row 2) algorithms from simulated data of the phantom generated with symmetrically truncated configurations A (a), B (b), C (c), D (d), E (e), and F(f) listed in table 1. Display window: [0.15, 0.3] cm^−1^.

Images of the digital abdomen phantom reconstructed within the phantom support by use of the TV-L1 and FBP algorithms are displayed, respectively, in rows 1 and 2 of figure 5. Again, it can be seen that the TV-L1 algorithm yields visually accurate images within the support of the digital abdomen phantom for all of the symmetrically truncated configurations listed in table 1 and that significant artifacts observed in the corresponding FBP reconstructions, as shown in row 2 of figure 5, are corrected for in images of the TV-L1 algorithm.

#### 3.3.2. Quantitative evaluation of the images reconstructed

**Figure 6.**
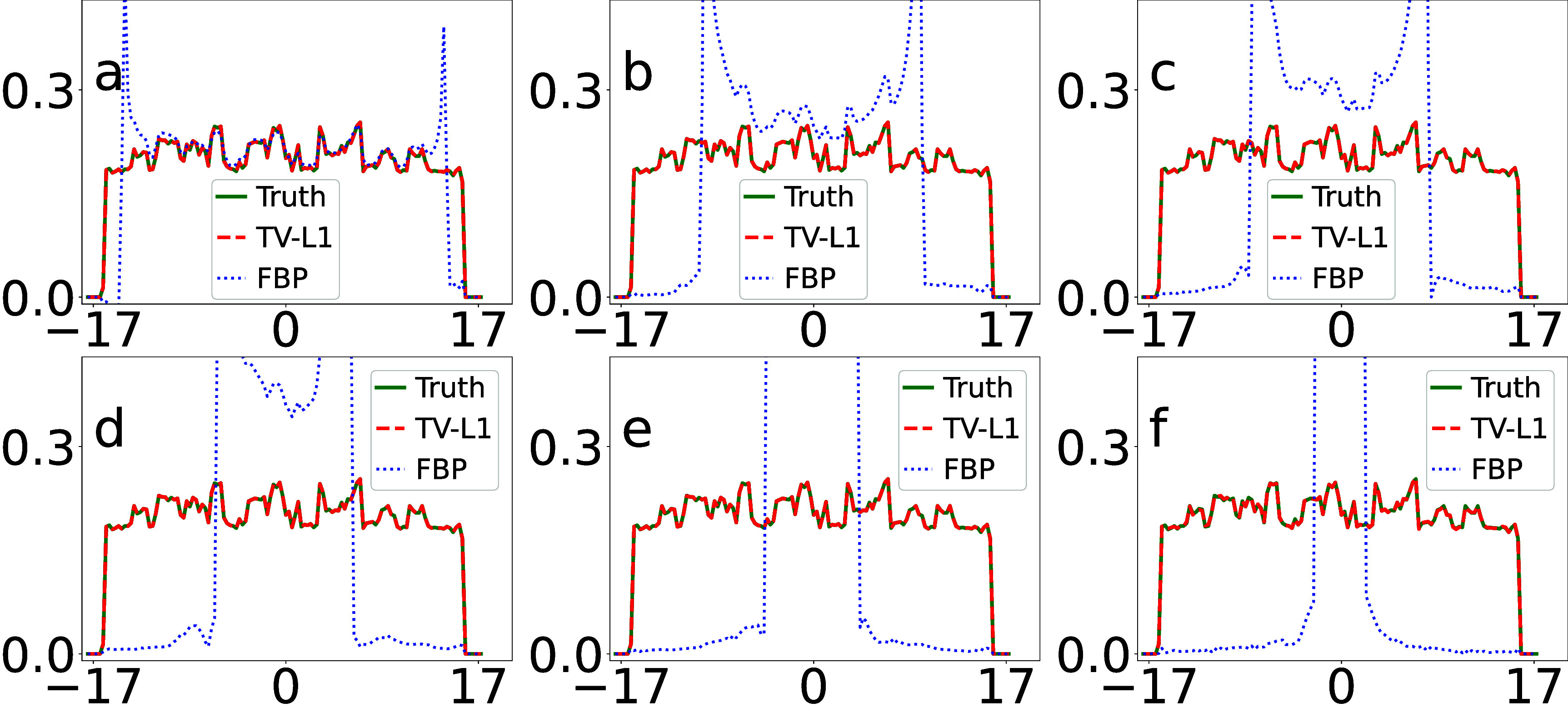
Image profiles (a)–(f) of the digital abdomen phantom in figure 5 over the white line indicated in figure 2(d), reconstructed by use of the TV-L1 (dashed) and FBP (dotted) algorithms, along with the corresponding truth image profiles (solid) of the digital abdomen phantom. The abscissas in the plots are of unit cm. It can be observed that the image-profile values of the FBP reconstructions can be beyond the maximum value used in the plot.

We display in figure 6 the profiles of the images in figure 5 over the white horizontal line highlighted in figure 2(d) for revealing quantitatively image-reconstruction accuracy. It can be observed that the image profiles of the TV-L1 reconstruction from data with truncation agree well with their respective truth image profile of the digital abdomen phantom, correcting for significant biases of image profiles in the FBP reconstructions.

We compute metrics nRMSE and PCC from images of the digital abdomen phantom in figure 5 and display them in figures B2 and B4, which reveal that as the level of data truncation increases, while metric nRMSE increases moderately, metric PCC remains largely 1, indicating numerically accurate image reconstruction of the digital abdomen phantom.

### 3.4. Image reconstruction for asymmetrically truncated configurations

**Figure 7.**
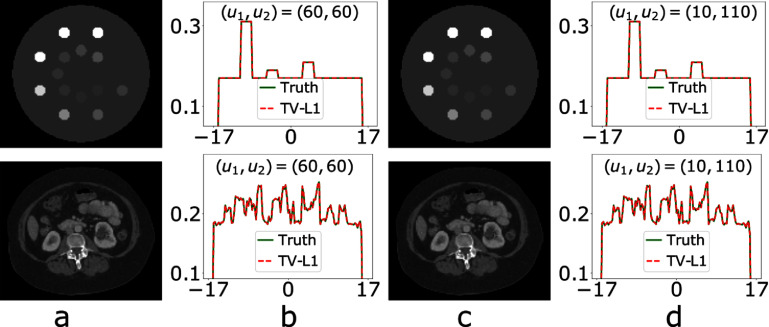
Images (columns a and c) and profiles (columns b and d) of the digital DE phantom (row 1) and digital abdomen phantom (row 2) reconstructed within the respective phantom supports by use of the TV-L1 algorithm for a symmetrically truncated configuration of $(u_1, u_2) = (60, 60)$ (columns a and b) and for an asymmetrically truncated configuration of $(u_1, u_2) = (10, 110)$ (columns c and d). Display windows: [0.1, 0.3] cm^−1^ for row 1 and [0.15, 0.3] cm^−1^ for row 2; and the abscissas in the plots are of unit cm. The truth image profiles (solid) of the digital DE and abdomen phantoms are plotted in columns (b) and (d).

The symmetrically and asymmetrically truncated configurations of detectors of identical length have FOVs of different sizes, as shown in figures 1(b) and (c), and the FOV of the latter is larger than that of the former. We have performed studies using simulated data of the digital DE and abdomen phantoms generated with symmetrically and asymmetrically truncated configurations of identical detector length $u_1+u_2$. For a symmetrically truncated configuration of $(u_1, u_2) = (60, 60)$ (i.e. symmetrically truncated configuration D in table 1) and an asymmetrically truncated configuration of $(u_1, u_2) = (10, 110)$, we select $\mathcal{M}_o$ and compute *l* and *t* from the digital DE and abdomen phantoms, as described in section 3.1 above. We then reconstruct the images of the digital DE and abdomen phantoms from their respective data generated. In figure 7, we display the images reconstructed within the phantom supports, along with the respective image profiles over the white lines depicted in figures 2(a) and (d). In this case of simulated data, the results reveal that the TV-L1 algorithm yields images of an identical level of numerical accuracy in terms of visual inspection and image profile for both symmetrically and asymmetrically truncated configurations.

## 4. Real-data studies

The simulated-data study above verifies the TV-L1 algorithm and reveals its potential (i.e. performance upper bound) to invert the truncated data model (i.e. equation (1)) for image reconstruction within the subject support that is substantially larger than the FOV. We investigate below the stability of the TV-L1 algorithm using real data that contain physical factors, including noise, scatter, and beam-hardening, that are not considered in the truncated data model in equation (1).

### 4.1. Experimental design of the real-data studies

#### 4.1.1. Subjects scanned

**Figure 8.**
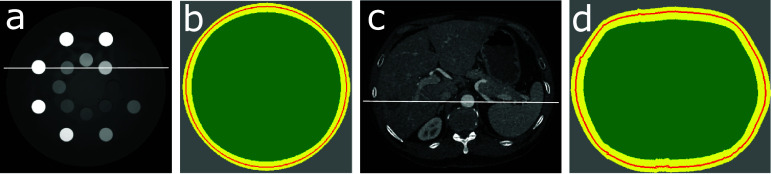
(a) and (c): Images of the physical DE and clinical abdomen phantoms reconstructed within their respective regions of $\mathcal{M}_i$ by use of the TV-L1 algorithm from their respective full data without truncation, display window: [0.15, 0.3] cm^−1^; and schematics (b) and (d) of annulus regions $\mathcal{M}_o $ (yellow) containing the exterior boundaries (red contours) and $\mathcal{M}_i $ regions (green) estimated from images in (a) and (c). Image profiles are plotted over the white lines depicted in (a) and (c) in the real-data studies below.

In the real-data studies, we use the physical DE (JRTAssociates 2015) and clinical abdomen phantom of distinctly different anatomies, as shown in figures 8(a) and (c). The former is used widely for CT imaging calibration, whereas the latter mimics clinically realistic anatomy of human abdomen. In the real-data studies, images are reconstructed on 2D discrete arrays of $512\times 512$ and $600\times 600$ square pixels of sizes 0.68 mm and 0.78 mm, respectively, for the physical DE and clinical abdomen phantoms.

#### 4.1.2. Scanning configuration geometry

A CT scanner in axial mode is used to scan a subject at 1200 views evenly distributed over 360^∘^; and the x-ray source and the central-row detector form an equivalent fan-beam geometry of SRD and SDD of 60 cm and 107 cm. The curved central-row detector consists of 896 detector bins with equal-angular separations of interval 0.055^∘^, thus forming an equal-angular fan-beam geometry of 49.2^∘^. For the physical DE and clinical abdomen phantoms considered, 620 and 660 detector bins in the central portions of the curved central-row detector, forming fan-beam angles of 34.04^∘^ and 36.24^∘^, respectively, yield FOVs that completely, but tightly, enclose the supports of the physical DE phantom and clinical abdomen phantom. Therefore, we use the central portions, i.e. $2U = 620$ or $2U = 660$ detector bins, of the central-row detector to collect the full data without truncation from the physical DE phantom and the clinical abdomen phantom, respectively. Subsequently, the symmetrically truncated configurations are formed, respectively, by use of only the central portions of the $2U = 620$ or $2U = 660$ detector bins, as listed in tables 2 and 3, for the physical DE and clinical abdomen phantoms.

**Table 2.** Symmetrically truncated configurations in real-data studies of the physical DE phantom, with $2U = 620$ detector bins.

Configurations	$(u_1$, $u_2)$	$u_1+u_2$	$(u_1+u_2)/2U$
A	(250, 250)	500	80.6%
B	(200, 200)	400	64.5%
C	(150, 150)	300	48.4%
D	(100, 100)	200	32.3%
E	(60, 60)	120	19.4%
F	(30, 30)	60	9.7%

**Table 3.** Symmetrically truncated configurations in real-data studies of the clinical abdomen phantom, with $2U = 660$ detector bins.

Configurations	$(u_1$, $u_2)$	$u_1 + u_2$	$(u_1+u_2)/2U$
A	(275, 275)	550	83.3%
B	(250, 250)	500	75.8%
C	(200, 200)	400	60.6%
D	(175, 175)	350	53.3%
E	(150, 150)	300	45.5%
F	(100, 100)	200	30.3%

#### 4.1.3. Data collection

As the FOV considered is sufficient to cover completely the support of the physical DE or clinical abdomen phantom, full data are collected without truncation. Data of the physical DE and clinical abdomen phantoms are of noise levels lower than and comparable to that of a typical clinical CT scan. From full data of the physical DE or clinical abdomen phantom collected, we subsequently extract real data with truncation of varying levels at each of 1200 views for symmetrically truncated configurations listed, respectively, in tables 2 and 3.

#### 4.1.4. Selection of the constraint parameters

In the simulated-data study, knowledge of the image support and exterior boundary of the digital phantom is known from which truth constraint parameters $\mathcal{M}_o$, *l*, and *t* can be obtained, as described in section 3.1. However, in a real-data study, the image support and exterior boundary of the subject scanned, and consequently the ‘optimal’ constraint parameters, are generally unknown. In this case, the appropriate selection (i.e. tuning) of the constraint parameters depends upon a number of factors (Zhang *et al* 2016), including data quality (e.g. levels of noise, scatters, and beam-hardening) and especially the goodness metric chosen for guiding the parameter selection, as discussed below.

We take an approach (Bian *et al* 2014, Han *et al* 2015) to empirically selecting the ‘optimal’ constraint parameters tuned to a real-data study: we perform multiple image reconstructions by use of the TV-L1 algorithm from the same set of real data with truncation by using multiple sets of $\mathcal{M}_o$, *l*, and *t* chosen; and we then estimate qualitatively or compute quantitatively multiple values of the goodness metric chosen from the multiple images reconstructed. The set of $\mathcal{M}_o$, *l*, and *t* that yield the image with the empirically ‘highest’ value of the goodness metric is chosen thus as the ‘optimal’ constraint parameters for the real-data study.

Specifically, in each of the real-data studies below, image visual inspection is used as the qualitative goodness metric for choosing $\mathcal{M}_o$, *l*, and *t* in terms of images with visually minimal truncation artifacts. Clearly, even if the ‘optimal’ selections of $\mathcal{M}_o$, *l*, and *t* for different real-data studies are based upon an identical goodness metric, they can be different as they are selected specific to the respective real-data studies involving data with truncation of varying degrees and/or physical factors, such as noise and scatters, of varying levels. In each of the real-data studies, we have indeed conducted multiple image reconstructions with multiple selections of $\mathcal{M}_o$, *l*, and *t* and include in sections 4.2 and 4.3 below the results obtained with empirically ‘optimal’ $\mathcal{M}_o$, *l*, and *t* selected based upon visual inspection of images reconstructed. Conversely, images reconstructed with other ‘non-optimal’ selections of $\mathcal{M}_o$, *l*, and *t* are not included here because they yield no new knowledge of significance.

We note again that this approach to selecting (i.e. tuning) the constraint parameters is used widely in research and application studies involving real data. For example, in a real-data study involving data with truncation, even if little or no knowledge is available about the exterior boundary of the subject imaged, multiple $\mathcal{M}_o$, along with *l* and *t*, can be selected for image reconstruction, and the empirically ‘optimal’ selection of $\mathcal{M}_o$ thus specifies the ‘exterior’ boundary of the image tailored to data with truncation in the real-data study, as the examples shown in figures 8(b) and (d) that correspond to the empirically ‘optimal’ selections of $\mathcal{M}_o$ for the two real-data studies below. (We note again that the annulus regions (yellow) of widths larger than the three-pixel width are displayed schematically in figures 8(b) and (d) for clearly revealing the exterior boundary (red) of the physical DE or clinical abdomen phantoms chosen in a real-data study.)

With the selection of annulus region $\mathcal{M}_o$, region $\mathcal{M}_i$ is also determined, as it is inscribed in the interior boundary of the annulus region of three-pixel width of $\mathcal{M}_o$, and it is thus as large as the image support excluding the region sandwiched between the exterior boundary of the image support and the interior boundary of region $\mathcal{M}_i$. Therefore, in the real-data studies, the regions of $\mathcal{M}_i$ are only slightly smaller than the subject supports, but substantially larger than the FOVs of the symmetrically truncated configurations, including those of significant truncation levels listed in tables 2 and 3.

#### 4.1.5. Image analysis

In each of the real-data studies below, the final image is reconstructed when the necessary convergence conditions in appendix B are reached numerically in terms of the single-precision floating-point error (Chambolle and Pock 2011, Sidky *et al* 2012, Zhang *et al* 2021). We perform visual inspection of the image reconstructed within region $\mathcal{M}_i$, followed by a quantitative analysis of image profiles reconstructed within the region. Using the image of the physical DE or clinical abdomen phantom reconstructed full data without truncation by use of the TV-L1 algorithm in figures 8(a) or (c) as the reference image in equations (B.3) and (B.5), we also compute quantitative metrics nRMSE and PCC within region $\mathcal{M}_i$.

### 4.2. Results of the physical DE phantom

In a real-data study of the physical DE phantom for a symmetrically truncated configuration listed in table 2, we empirically choose ‘optimal’ $\mathcal{M}_o$, *l*, and *t*, as described in section 4.1 and then use them in the TV-L1 algorithm to reconstruct the image from data of the physical DE phantom collected by use of the symmetrically truncated configuration. As no knowledge of the ‘truth image’ of the physical DE phantom is available, we choose a reference image that is reconstructed from full data without truncation by use of the TV-L1 algorithm, as shown in figure 8(a).

#### 4.2.1. Visual inspection of the images reconstructed

**Figure 9.**
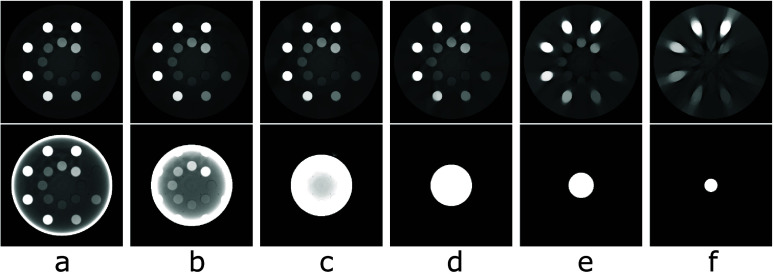
Images of the physical DE phantom reconstructed within their respective regions of $\mathcal{M}_i$ by use of the TV-L1 (row 1) and FBP (row 2) algorithms from real data collected with symmetrically truncated configurations A (a), B (b), C (c), D (d), E (e), and F (f) listed in table 2. Display window: [0.15, 0.3] cm^−1^.

Using the constraint parameters selected, we reconstruct images of the physical DE phantom by using the TV-L1 algorithm, and display images within region $\mathcal{M}_i$ in row 1 of figure 9, along with the respective FBP reconstructions in row 2 of figure 9. It can be observed in figure 9 that the TV-L1 algorithm yields visually images with much reduced artifacts within regions of $\mathcal{M}_i$ substantially larger than the FOV and that the significant truncation artifacts in the FBP reconstructions are corrected effectively for in the images of the TV-L1 algorithm.

#### 4.2.2. Quantitative evaluation of the images reconstructed

**Figure 10.**
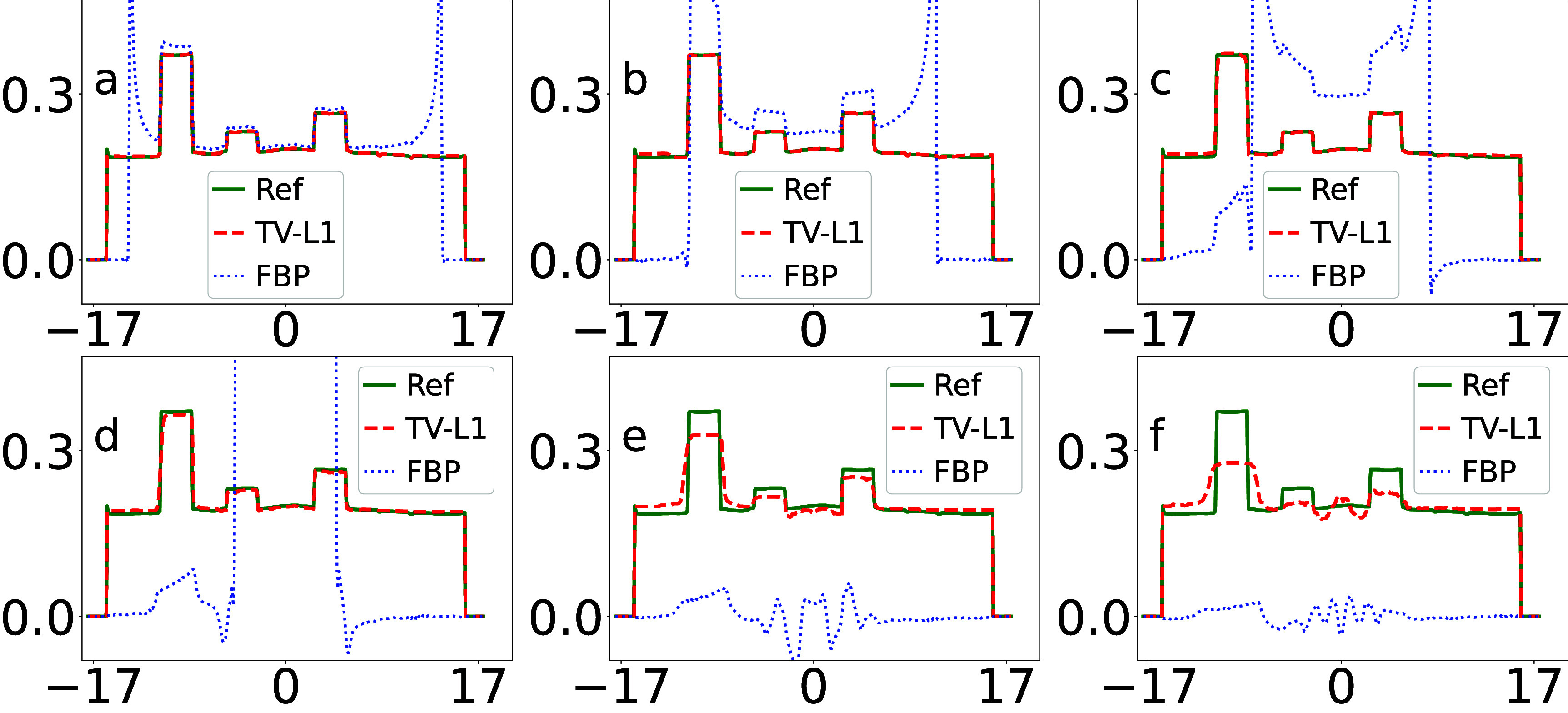
Image profiles (a)–(f) of the physical DE phantom in figure 9 over the white line indicated in figure 8(a), reconstructed by use of the TV-L1 (dashed) and FBP (dotted) algorithms, along with the corresponding reference image profiles (solid) of the physical DE phantom. The abscissas in the plots are of unit cm. It can be observed that the image-profile values of the FBP reconstructions can be beyond the maximum value used in the plot.

**Figure B3.**
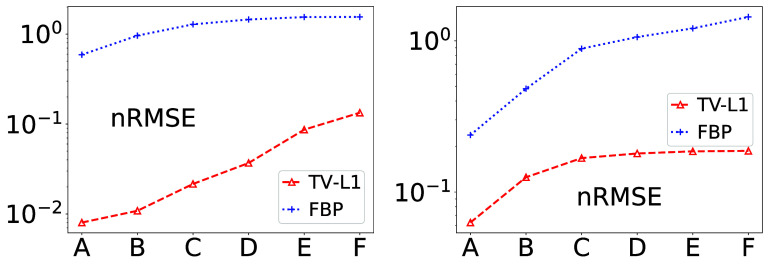
Metric nRMSE calculated from the images of the physical DE phantom (left) and clinical abdomen phantom (right) reconstructed by use of the TV-L1 (dashed) and FBP (dotted) algorithms.

**Figure B4.**
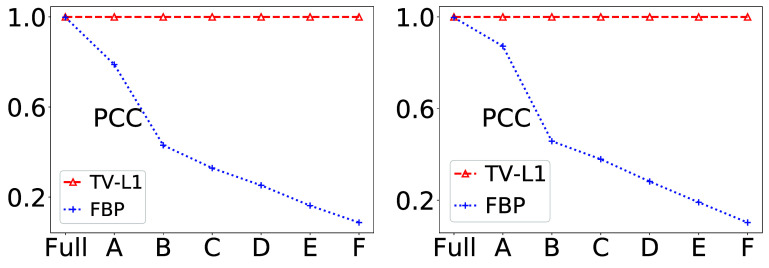
Metric PCC calculated from the images of the digital DE phantom (left) and digital abdomen phantom (right) reconstructed by use of the TV-L1 (dashed) and FBP (dotted) algorithms.

**Figure B5.**
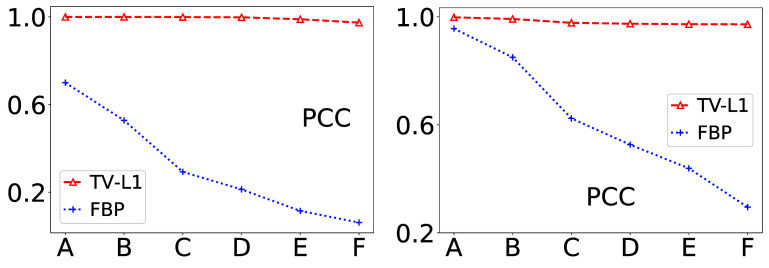
Metric PCC calculated from the images of the physical DE phantom (left) and clinical abdomen phantom (right) reconstructed by use of the TV-L1 (dashed) and FBP (dotted) algorithms.

We plot in figure 10 the profiles of the images in figure 9 over the white horizontal line highlighted in figure 8(a). It can be observed that the image profiles of the TV-L1 reconstruction from data with truncation of varying degrees agree quantitatively well with the respective reference image profiles. Using the reference image of the physical DE phantom in figure 8(a), we compute metrics nRMSE and PCC from images in figure 9 reconstructed by use of the TV-L1 algorithm, and display them in figures B3 and B5, which reveal that as the level of data truncation increases, while metric nRMSE increases moderately, metric PCC largely remains 1, indicating stable, and reasonably accurate, image reconstruction of the physical DE phantom within region $\mathcal{M}_i$. Metrics nRMSE and PCC computed from the FBP reconstructions in figure 9 worsen rapidly as the level of data truncation increases.

### 4.3. Results of the clinical abdomen phantom

Similar to the case of the physical DE phantom in section 4.2, in a real-data study of the clinical abdomen phantom for a symmetrically truncated configuration listed in table 3, we empirically select ‘optimal’ $\mathcal{M}_o$, *l*, and *t*, as described in section 4.1 and then use them in the TV-L1 algorithm to reconstruct the image from data of the clinical abdomen phantom collected with the symmetrically truncated configuration. Again, in real-data studies, no knowledge of the ‘truth image’ of the clinical abdomen phantom is available. Therefore, we choose a reference image reconstructed from full data without truncation by use of the TV-L1 algorithm, as shown in figure 8(c).

#### 4.3.1. Visual inspection of the images reconstructed

**Figure 11.**
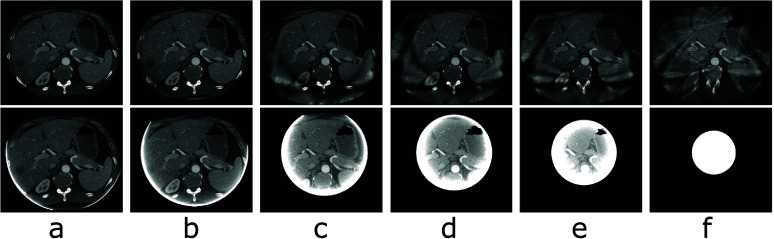
Images of the clinical abdomen phantom reconstructed within their respective regions of $\mathcal{M}_i$ by use of the TV-L1 (row 1) and FBP (row 2) algorithms from real data collected with symmetrically truncated configurations A (a), B (b), C (c), D (d), E (e), and F (f) listed in table 3. Display window: [0.15, 0.3] cm^−1^.

Using the constraint parameters selected for data collected with each of the symmetrically truncated configurations listed in table 3, we reconstruct images of the clinical abdomen phantom by using the TV-L1 algorithm, and display images within region $\mathcal{M}_i$ in row 1 of figure 11, along with the respective FBP images in row 2 of figure 11. It can be observed that the TV-L1 algorithm yields images with reduced artifacts within a region substantially larger than the FOV, in which significant truncation artifacts in the FBP reconstructions are reduced.

#### 4.3.2. Quantitative evaluation of the images reconstructed

**Figure 12.**
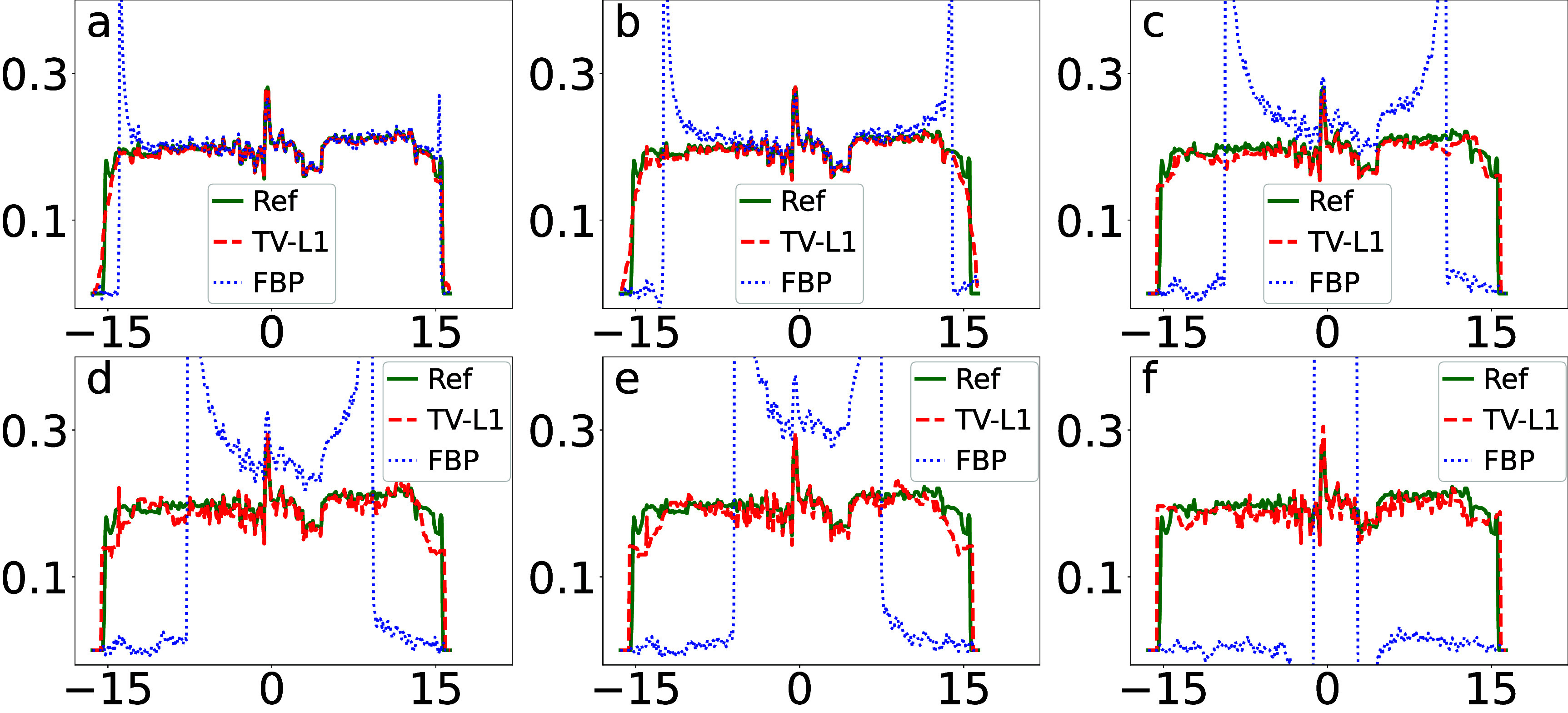
Image profiles (a)–(f) of the clinical abdomen phantom in figure 11 over the white line indicated in figure 8(c), reconstructed by use of the TV-L1 (dashed) and FBP (dotted) algorithms, along with the corresponding reference image profiles (solid) of the clinical abdomen phantom. The abscissas in the plots are of unit cm. It can be observed that the image-profile values of the FBP reconstructions can be beyond the maximum value used in the plot.

We plot in figure 12 the profiles of the images in figure 11 over the white horizontal line highlighted in figure 8(c). It can be observed that the image profiles of the TV-L1 reconstruction from data with truncation of varying degrees agree quantitatively well with the respective reference image profiles. Using the reference image of the clinical abdomen phantom in figure 8(c), we compute metrics nRMSE and PCC from images in figure 11 reconstructed by use of the TV-L1 algorithm, and plot them in figures B3 and B5. It can be observed that as the level of data truncation increases, while metric nRMSE increases, metric PCC largely remains 1, indicating image reconstruction of the clinical abdomen phantom is with reduced artifacts within region $\mathcal{M}_i$. Metrics nRMSE and PCC computed from the FBP reconstructions in figure 11 worsen rapidly as the level of data truncation increases.

### 4.4. Image reconstruction for asymmetrically truncated configurations

**Figure 13.**
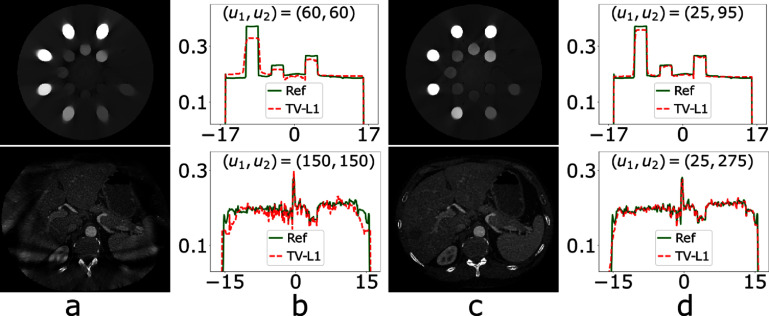
Images (columns a and c) and profiles (columns b and d) of the physical DE phantom (row 1) and clinical abdomen phantom (row 2) reconstructed within their respective regions of $\mathcal{M}_i$ by use of the TV-L1 algorithm for a symmetrically (columns a and b) and asymmetrically (columns c and d) truncated configurations, display window: [0.15, 0.3] cm^−1^. The symmetrically truncated configuration is of $(u_1, u_2) = (60, 60)$ (columns a and b) for the physical DE phantom (row 1) and of $(u_1, u_2) = (150, 150)$ (columns a and b) for the clinical abdomen phantom (row 2); whereas the asymmetrically truncated configuration is of $(u_1, u_2) = (25, 95)$ (columns c and d) for the physical DE phantom (row 1) and of $(u_1, u_2) = (25, 275)$ (columns c and d) for the clinical abdomen phantom (row 2). The abscissas in the plots are of unit cm. The reference image profiles (solid) of the physical DE phantom and clinical abdomen phantom are plotted also in columns (b) and (d).

We have performed real-data studies of the physical DE and clinical abdomen phantoms by using data collected with symmetrically and asymmetrically truncated configurations of identical detector length $u_1+u_2$. Specifically, from real data of the physical DE phantom collected with a symmetrically truncated configuration of $(u_1, u_2) = (60, 60)$ (i.e. symmetrically truncated configuration E in table 2) and an asymmetrically truncated configuration of $(u_1, u_2) = (25, 95)$, we select (i.e. tune) constraint parameters $\mathcal{M}_o$, *l*, and *t* as described in section 4.1, reconstruct images by use of the TV-L1 algorithm, and display the images within the respective regions of $\mathcal{M}_i$ in row 1 of figure 13, along with their corresponding profiles over the white lines depicted in figure 8(a). Using the reference image of the physical DE phantom, we also compute metrics nRMSE and PCC from the images in row 1 of figures 13(a) and (c), and observe that nRMSE and PCC of the asymmetrically truncated configuration are better than those of the symmetrically truncated configuration.

Moreover, from real data of the clinical abdomen phantom collected with a symmetrically truncated configuration of $(u_1, u_2) = (150, 150)$ (i.e. symmetrically truncated configuration E in table 3) and an asymmetrically truncated configuration of $(u_1, u_2) = (25, 275)$, we then reconstruct the images by use of the TV-L1 algorithm and display the images within the respective regions of $\mathcal{M}_i$ in row 2 of figure 13, along also with their respective profiles over the white lines depicted in figure 8(c). Again, using the reference image of the clinical abdomen phantom, we compute metrics nRMSE and PCC from the images in row 2 of figures 13(a) and (c), and observe that nRMSE and PCC of the asymmetrically truncated configuration are better than those of the symmetrically truncated configuration.

It can be observed that in real data cases for the asymmetrically and symmetrically truncated configurations of identical detector lengths, the former yields visually (i.e. the images) and numerically (i.e. the image profiles and metrics nRMSE and PCC) more accurate results than does the latter. This observation suggests that in real-data studies, for a given detector length, the use of an asymmetrically truncated configuration may be preferred over that of a symmetrically truncated configuration. We have conducted additional real-data studies for asymmetrically truncated configurations of a variety of ratios $u_1/u_2$ and obtain results similar to those in the cases discussed.

## 5. Discussion

In the work, we investigate and develop the TV-L1 algorithm that effectively inverts the truncated data model for numerically accurate image reconstruction within the subject support, or within the region of size comparable to the subject support yet substantially larger than the truncated scanning FOV, in fan-beam CT. Observing that data truncation leads to an unconventional, pixel-varying-LAR problem, we formulate image reconstruction from data with truncation as an optimization program with hybrid, region-based image constraints, including a unique $\ell_1$-norm constraint in effect on the image’s exterior boundary for effective suppression of truncation artifacts in image reconstructed. The TV-L1 algorithm is developed and evaluated subsequently to solve the optimization program for yielding a numerically accurate image within the subject support or a region slightly smaller than the subject support yet substantially larger than the scanning FOV.

The existing directional-TV (DTV) algorithm has been demonstrated to numerically accurately and stably reconstruct images from data without truncation collected only over a LAR in x-ray CT (Zhang *et al* 2021); and it employs DTV constraints on the image along unique, LAR-dependent directions for effectively suppressing LAR artifacts observed otherwise in images reconstructed by use of other algorithms. The reason for the effectiveness of the DTV algorithm is that unique directions can be selected, along which the DTV constraints are devised, because the directions of x-rays passing through any point within the image support span an identical LAR. As discussed in appendix A, however, in the pixel-varying-LAR problem of data truncation, the extents and directions of the LARs spanned by the x-rays passing through the pixels outside the scanning FOV vary, depending upon the specific locations of the pixels. Therefore, no unique directions can be identified for devising image DTVs in the pixel-varying-LAR problem of data truncation. Instead, the region-based constraint on the image $\ell_1$-norm within a annulus region containing the exterior boundary of the image is devised for effectively suppressing truncation artifacts in images reconstructed.

We conduct numerical studies that reveal and characterize the accuracy and stability of the TV-L1 algorithm using simulated and real data of varying truncation degrees from subjects of anatomic complexity of varying levels. The simulated-data studies show that the TV-L1 algorithm can yield numerically accurate images within the supports of digital phantoms studied. Meanwhile, the real-data studies demonstrate that the TV-L1 algorithm can stably yield numerically accurate images within the region of size comparable to the subject support and thus substantially larger than the scanning FOV from data with truncation of varying degrees.

A detector of length shorter than one half of the full detector length can be arranged to create a symmetrically or asymmetrically truncated configuration, as shown in figures 1(b) and (c); and the latter has an effective FOV larger than that of the former. The simulated-data study indicates that, for the digital DE and abdomen phantoms considered, the TV-L1 algorithm can reconstruct numerically accurate images within the support of each of the digital phantoms from data of the symmetrically and asymmetrically truncated configurations of identical detector lengths.

In real-data studies of the symmetrically and asymmetrically truncated configurations of identical detector lengths, however, as real data containing physical factors inconsistent with the data model in equation (2), the TV-L1 algorithm reconstruct images from real data of the asymmetrically truncated configuration more numerically accurately than from real data of the symmetrically truncated configuration. Therefore, if the practical workflow permits, an asymmetrically truncated configuration would be more favorable than a symmetrically truncated configuration.

We note that annulus region $\mathcal{M}_o$ concerning the support or exterior boundary of the subject imaged is considered a constraint parameter instead of prior knowledge. In a real-data study, like constraint parameters *l* and *t*, $\mathcal{M}_o$ is determined (or tuned) with the guidance of a goodness metric chosen that is of relevance to the study task, as described in section 4.1. While $\mathcal{M}_o$, *l*, and *t* are considered a part of the design of the image reconstruction, they are not considered prior knowledge. Instead, some prior knowledge about them, if available, can certainly be exploited for helping tune the constraint parameters specific to the (e.g. real data) study. For example, if the subject support can be obtained in a prior scan without truncation, then one can perform multiple reconstructions with multiple $\mathcal{M}_o$ selected in the neighborhood of the subject support, thus possibly reducing the search space for $\mathcal{M}_o$ in the real-data study.

**Figure A1.**
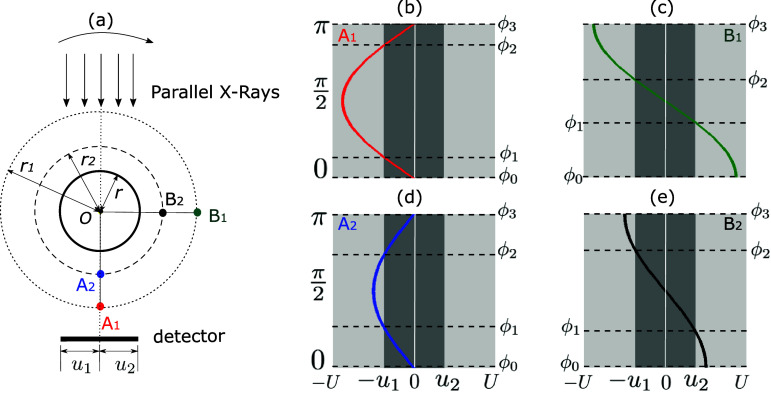
(a): A parallel-beam geometry with a FOV enclosed by the circle (thick solid) of radius *r*, centered at COR *O*. Pixels on the two circles with radii *r*_1_ (dotted) and *r*_2_ (dashed) are outside the FOV and thus are truncated at some projection views, where $r < r_2 < r_1$. Pixels at *A*_1_ and *B*_1_ and at *A*_2_ and *B*_2_, respectively, situate on the two circles; whereas points *O*, *A*_1_, and *A*_2_ are colinear, so are points *O*, *B*_1_, and *B*_2_. (b), (c), (d), and (e): data spaces of the parallel-beam geometry in each of which the horizontal and vertical axes denote, respectively, the full angular range of *π* and full detector length of 2*U*; the loci of points *A*_1_, *B*_1_, *A*_2_, and *B*_2_ are highlighted schematically by the red, green, blue, and black curves, respectively; and *φ*_0_ = 0 and $\phi_3 = \pi$, whereas *φ*_1_ and *φ*_2_ denote the views at which the loci, respectively, intersect with the edges of the vertical band (dark-grey) of the FOV.

In the work, we have considered image reconstruction from data with truncation collected by using a scanning FOV whose center coincides with the COR of the scanning system. In other words, the FOV is formed by use of detectors of lengths *u*_1_ and *u*_2_ unchanged for all of the views, and its loci makes a central vertical band in the data space, similar to the central vertical band (dark-grey) displayed in figure A1 for the parallel-beam geometry. However, a FOV can also be formed, e.g. with *u*_1_ and *u*_2_ changing from view to view, and thus the FOV center does not coiFncide with the COR of the scanning system. In this case, the loci of the FOV from all of the views can thus make, e.g. a band of sinusoidal shape in the data space. The optimization program and TV-L1 algorithm developed can readily be applied to addressing image reconstruction from data with truncation collected by use of such a scanning FOV.

Furthermore, the insights, analysis, and algorithm developed in the work can be generalized to address image reconstruction from data with truncation in cone-beam CT and possibly in dual-energy or photon-counting CT (Buzug 2008, Tang 2023), and other tomographic imaging modalities.

## 6. Conclusion

We develop the TV-L1 algorithm to invert effectively the truncated data model for numerically accurate and stable image reconstruction in fan-beam CT. Numerical studies using simulated and real data reveal that the TV-L1 algorithm can invert the truncated data model for numerically accurate and stable image reconstruction within the subject support or a region slightly smaller than the subject support but substantially larger than the scanning FOV, from data with truncation of varying degrees. The work can be of theoretical interest, as it yields insights into, and algorithms for, accurate and stable image reconstruction from data with truncation; and it may also be of practical implication for the development of heuristic procedures tailored effectively to address specific needs of accurate image reconstruction within a region of size comparable to that of the subject support that is substantially larger than the scanning FOV, from data with truncation possibly in clinical, biomedical, and industrial applications.

## Figures and Tables

**Figure B1.**
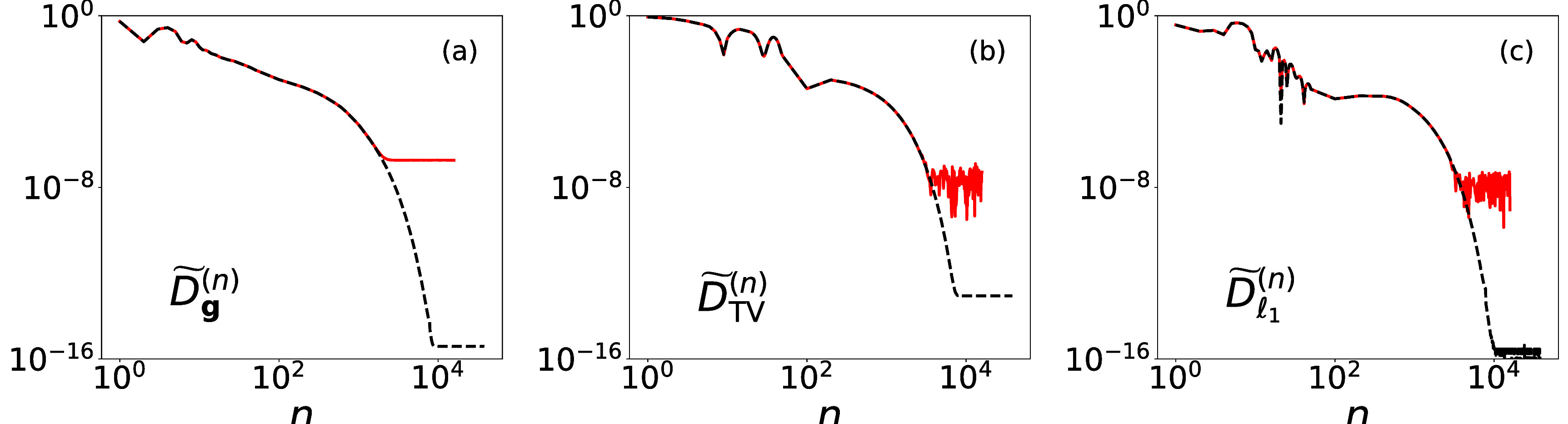
Convergence metrics (a) $\widetilde{D}^{(n)}_\mathbf{g}$, (b) $\widetilde{D}^{(n)}_\textrm{TV}$, and (c) $\widetilde{D}^{(n)}_{\ell_1}$ of the TV-L1 algorithm in reconstruction from full data of the digital abdomen phantom using single-precision (red solid) and double-precision (black dashed) floating-point errors, as functions of iteration number *n* (in log-log scales).

## Data Availability

The data cannot be made publicly available upon publication because the cost of preparing, depositing and hosting the data would be prohibitive within the terms of this research project. The data that support the findings of this study are available upon reasonable request from the authors.

## Appendix A Analysis of the root cause of truncation artifacts

We analyze the root cause of truncation artifacts in images reconstructed from data with truncation. For simplicity and clarity, we consider a continuous-to-continuous data model with a parallel-beam geometry in which x-rays are depicted by the parallel arrow-headed lines in figure A1(a), with the understanding that the analysis and insights gained can readily be extended to fan- and cone-beam geometries.

From the perspective of the inverse Radon transform, a necessary condition for a pixel value to be accurately reconstructable is that knowledge of the Radon transform is available for the measurable rays passing through the pixel from directions spanning the full angular range of 180^∘^. On the other hand, if knowledge of the Radon transform is available for the measurable rays passing through the pixel from directions spanning only a LAR of $ < 180^\circ$, it leads to a LAR problem for the pixel.

For analysis clarity without loss of generality, we consider a symmetrically truncated configuration, i.e. $u_1 = u_2$. As shown in figure A1(a), a detector of length $u_1 + u_2 $ thus forms a FOV enclosed by the thick-black circle of radius $r = u_1$ centered on point *O*, i.e. the COR of the scanning configuration. We consider two pairs of pixels that locate, respectively, at $\textrm{A}_1$ and $\textrm{B}_1$ on the circle of radius *r*_1_ (dotted) and at $\textrm{A}_2$, and $\textrm{B}_2$ on the circle of radius *r*_2_ (dashed), where $r < r_2 < r_1$.

Each of figures A1(b)–(e) denotes the parallel-beam projection space (i.e. the data space) in which the vertical axis denotes the projection angles ranging from 0 to 180^∘^, whereas the horizontal axis depicts the detector-bin positions. The loci of the pixels within the FOV are contained within the vertical bands (dark-grey) in the data space, whereas the red, green, blue, and black curves in figures A1(b)–(e) depict, respectively, the loci of pixels at $\textrm{A}_1$, $\textrm{B}_1$, $\textrm{A}_2$, and $\textrm{B}_2$ in figure A1(a).

For the pixels at A_1_ and A_2_ outside the FOV, only the portions (i.e. specified by $\phi_1-\phi_0$ and $\phi_3-\phi_2$) of their loci within the band (dark-grey) can be measurable, whereas portions (i.e. specified by $\phi_2-\phi_1$) outside the band (dark-grey) are not measurable, as shown in figures A1(b) and (d). Similarly, for the pixels at B_1_ and B_2_ outside the FOV, only the portions (i.e. specified by $\phi_2-\phi_1$) of their loci within the band (dark-grey) can be measurable, whereas portions (i.e. specified by $\phi_1-\phi_0$ and $\phi_3-\phi_2$) outside the band (dark-grey) are not measurable, as shown in figures A1(c) and (e).

The extents of the measurable or missing angular ranges of a pixel outside the FOV decrease or increase as the pixel is moving away from COR *O*. For the pixel pair at A_1_ and B_1_ or at A_2_ and B_2_ on the same circle of radius *r*_1_ or *r*_2_ outside the FOV, the extents of the portions of the loci measurable for A_1_ and B_1_, or for A_2_ and B_2_, are identical, i.e. the sum of $\phi_1-\phi_0$ and $\phi_3-\phi_2$ in (b) or (d) equals $\phi_2-\phi_1$ in (c) or (e), respectively. Therefore, a truncation problem is in essence tantamount to a pixel-varying-LAR problem yielding artifacts of some characteristics analogous to that observed in images reconstructed from LAR data.

## Appendix B Convergence conditions and image-quality metrics

### B.1. Necessary convergence conditions

For the TV-L1 algorithm, we consider dimensionless convergence metrics (Xia *et al* 2016, Zhang *et al* 2021)

\begin{equation*} \begin{split} \widetilde{D}^{\left(n\right)}_\mathbf{g} &amp; = \parallel \mathcal{W} \left(\mathcal{H} ^{\left(u_1, u_2\right)}\mathcal{M} \mathbf{f}^{\left(n\right)} - \mathbf{g}^{\left[\mathcal{M}\right]}\right) \parallel_2/||\mathbf{g}^{\left[\mathcal{M}\right]}||_2 \\ \widetilde{D}^{\left(n\right)}_\textrm{TV} &amp; = | \left(||\left(|\mathcal{M}_i^{\prime} \nabla \mathcal{M} \mathbf{f}^{\left(n\right)}|\right)||_1 - t\right)|/t \\ \widetilde{D}^{\left(n\right)}_{\ell_1} &amp; = | \left(||\mathcal{M}_o \nabla \mathcal{M} \mathbf{f}^{\left(n\right)}||_1 - l\right)|/l ,\\ \end{split}\end{equation*}

and necessary convergence conditions (Sidky *et al* 2012, Xia *et al* 2016, Zhang *et al* 2016)

\begin{equation*} \widetilde{D}^{\left(n\right)}_\mathbf{g}\rightarrow c, \,\,\,\,\,\, \widetilde{D}^{\left(n\right)}_\textrm{TV}\rightarrow 0, \,\,\,\,\,\,\textrm{and} \,\,\,\,\,\, \widetilde{D}^{\left(n\right)}_{\ell_1}\rightarrow 0,\end{equation*}

as $n\rightarrow \infty$, where *c* is a non-negative constant satisfying *c* = 0 for consistent data (e.g. simulated data) and *c* > 0 for inconsistent data (e.g. real data.) In each of the numerical studies in the work, the final image is reconstructed when the convergence conditions in equation (B.2) is reached numerically in terms of the single-precision floating-point error. We note that the convergence metrics are normalized to be dimensionless so that they fall into roughly comparable numerical ranges.

We plot in figure B1, as an example set, the convergence curves for the TV-L1 image reconstruction from full simulated-data of the digital abdomen phantom, which reveal the convergence behavior of the TV-L1 algorithm computed using both single-precision and double-precision floating-point errors. It can be observed that the TV-L1 algorithm can numerically and accurately converge the optimization problem in equation (3).

### B.2. Image-quality metric

We consider an image-quality metric, referred to as the normalized root-mean-square error (nRMSE), which is defined over a region selected within the subject support as

\begin{eqnarray*} \textrm{nRMSE}\left(\mathbf{f}^{\left(n\right)}\right) = ||\mathbf{f}^{\left(n\right)}-\mathbf{f}^\textrm{[ref]}||_2/||\mathbf{f}^\textrm{[ref]}||_2.\end{eqnarray*}

and then condition

\begin{equation*} \textrm{nRMSE}\left(\mathbf{f}^{\left(n\right)}\right) \rightarrow 0 \,\,\,\,\textrm{as} \,\,\,\,n\rightarrow \infty,\end{equation*}

where $\mathbf{f}^\textrm{(n)}$ and $\mathbf{f}^\textrm{[ref]}$ denote the image reconstructed at iteration *n* and a reference image to be chosen. We use $\mathbf{f}^{\star}$ to denote the converged reconstruction, as shown in equation (3) and in algorithm 1.

**Table:**

**Algorithm 1.** Pseudo-codes of the TV-L1 algorithm.
INPUT: $\mathbf{g}^{[\mathcal{M}]}$, $\mathcal{H}^{(u_1, u_2)}$, $\mathcal{M}_o$, *l*, *t*, and $\rho\approx 10^{-2}$
1: $\nu_1 \leftarrow \sqrt{e_K}/||\mathcal{M}^{\prime}_i \nabla \mathcal{M}||_2$, $\nu_2 \leftarrow \sqrt{e_K}/||\mathcal{M}_o \mathcal{M}||_2$, $\mu \leftarrow \sqrt{e_K}/||\mathcal{I}||_2$, Σ, $\mathcal{T}$
2: $n \leftarrow 0$
3: INITIALIZE: ${\mathbf{f}}^{(0)}$, $\mathbf{w}^{(0)}$, $\mathbf{p}^{(0)}$, $\mathbf{q}^{(0)}$, and $\mathbf{t}^{(0)}$ to zero
4: $\bar{\mathbf{f}}^{(0)} \leftarrow \mathbf{f}^{(0)}$
5: **repeat**
6: $\mathbf{w}^{(n+1)} = (\mathbf{w}^{(n)} + \Sigma \mathcal{W}(\mathcal{H}^{(u_1, u_2)}\mathcal{M}\bar{\mathbf{f}}^{(n)} - \mathbf{g}^{[\mathcal{M}]}))/(\mathcal{I}+\Sigma)$
7: $\bar{\mathbf{p}}^{(n)} = \mathbf{p}^{(n)} + \Sigma \nu_1 \mathcal{M}^{\prime}_i \nabla \mathcal{M} \bar{\mathbf{f}}^{(n)}$
$\bar{\mathbf{q}}^{(n)} = \mathbf{q}^{(n)} + \Sigma \nu_2 \mathcal{M}_o \mathcal{M} \bar{\mathbf{f}}^{(n)}$
8: $\mathbf{p}^{(n+1)} = \bar{\mathbf{p}}^{(n)} - \Sigma \frac{\bar{\mathbf{p}}^{(n)}}{|\bar{\mathbf{p}}^{(n)}|}\ell_1 \textrm{ball}_{\nu_1 t} (\frac{|\bar{\mathbf{p}}^{(n)}|}{\Sigma})$
$\mathbf{q}^{(n+1)} = \bar{\mathbf{q}}^{(n)} - \Sigma \frac{\bar{\mathbf{q}}^{(n)}}{|\bar{\mathbf{q}}^{(n)}|}\ell_1 \textrm{ball}_{\nu_2 l} (\frac{|\bar{\mathbf{q}}^{(n)}|}{\Sigma})$
$\mathbf{t}^{(n+1)} = \textrm{neg}({\mathbf{t}^{(n)} + \Sigma \mu \bar{\mathbf{f}}^{(n)}})$
9: $\mathbf{f}^{(n+1)} = \mathbf{f}^{(n)}-\mathcal{T}(\mathcal{M}^\top\mathcal{H}^{(u_1, u_2) \top}\mathcal{W}^{\top}\mathbf{w}^{(n+1)}+\nu_1 \mathcal{M}^\top\nabla^{\top}\mathcal{M}_i^{^{\prime}\top}{\mathbf{p}}^{(n+1)}$
$+ \nu_2 \mathcal{M}^\top \mathcal{M}_o ^{\top}{\mathbf{q}}^{(n+1)} + \mu \mathbf{t}^{(n+1)})$
10: $\bar{\mathbf{f}}^{(n+1)} = 2 \mathbf{f}^{(n+1)}-\mathbf{f}^{(n)}$
11: $n \leftarrow n+1$
12: **until** the necessary convergence conditions are satisfied
13: OUTPUT: final image $\mathbf{f}^\star = \mathbf{f}^{(n)}$

In the simulated-data study, we choose $\mathbf{f}^\textrm{[ref]} = \mathbf{f}^\textrm{[truth]}$, where $\mathbf{f}^\textrm{[truth]}$ denotes the truth image of a digital phantom, compute the metric over the subject support, and then use equation (B.4) as the necessary and sufficient condition on the numerically accurate inversion of the data model in equation (1) by use of the TV-L1 algorithm. We have computed the metric using $\mathbf{f}^\star$ reconstructed and truth image $\mathbf{f}^\textrm{[truth]}$ of the digital DE or abdomen phantom for each of the truncation configurations considered and plot it in figure B2.

In a real-data study, however, as $\mathbf{f}^\textrm{[truth]}$ is generally unknown or even undefined, we choose the image, reconstructed from full data without truncation, which is available in the work, as reference image $\mathbf{f}^\textrm{[ref]}$; and we then compute the metric with $\mathbf{f}^{(n)}$ and $\mathbf{f}^\textrm{[ref]}$ within region $\mathcal{M}_i$. In this case, even if equation (B.4) is satisfied, it is not a sufficient and necessary condition on the numerically accurate inversion of the data model in equation (1) by use of the TV-L1 algorithm; instead, it indicates only that $\mathbf{f}^{(n)}$ approaches $\mathbf{f}^\textrm{[ref]}$. We have computed the metric using $\mathbf{f}^\star$ reconstructed and reference image $\mathbf{f}^\textrm{[ref]}$ of the physical DE or clinical abdomen phantom for each of the truncation configurations considered and plot it in figure B3.

We also consider an additional image-quality metric, referred to as the Pearson-correlation coefficient (PCC), which measures the degree of similarity of converged image $\mathbf{f}^{\star}$ to a reference image:

\begin{eqnarray*} \textrm{PCC}\left(\mathbf{f}^{\star}\right) &amp; = &amp; \frac{\vert Cov\left(\mathbf{f}^{\star}, \mathbf{f}^\textrm{[ref]}\right)\vert}{\sigma\left(\mathbf{f}^{\star}\right) \, \sigma\left(\mathbf{f}^\textrm{[ref]}\right)},\end{eqnarray*}

where $\textrm{Cov}(\mathbf{f}^{\star}, \mathbf{f}^\textrm{[ref]})$ denotes the ‘spatial covariance’ between image $\mathbf{f}^{\star}$ reconstructed and reference image $\mathbf{f}^\textrm{[ref]}$ within a region selected; $\sigma^{2}(\mathbf{f}) = \textrm{Cov}(\mathbf{f}, \mathbf{f})$ the ‘spatial variances’ of **f**. For each of the truncation configurations, we compute the PCCs and plot them in figure B4 for the digital DE and abdomen phantoms and in figure B5 for the physical DE and clinical abdomen phantom.
